# P-1147. Mupirocin Resistance Among Methicillin-Resistant Staphylococcus aureus Surveillance Isolates Associated with Invasive Infections, 2005-2022

**DOI:** 10.1093/ofid/ofaf695.1341

**Published:** 2026-01-11

**Authors:** Holly Biggs, Davina Campbell, Kelly A Jackson, Megan Taylor, Shirley Zhang, Joelle Nadle, Susan M Ray, Jessica Howard-Anderson, Ruth Lynfield, Carmen Bernu, Ghinwa Dumyati, Marissa Walsh, William Schaffner, Tiffanie M Markus, Keipp Talbot, Joseph D Lutgring, Amy Gargis, Isaac See

**Affiliations:** CDC, Atlanta, GA; Division of Healthcare Quality Promotion, Centers for Disease Control and Prevention, Atlanta, GA; U.S. Centers for Disease Control and Prevention, Atlanta, Georgia; Centers for Disease Control and Prevention, Atlanta, Georgia; Center Disease of Control, Atlanta, Georgia; California Emerging Infections Program, Oakland, California; Emory University School of Medicine, Atlanta, Georgia; Emory University, Atlanta, GA; Minnesota Department of Health, St. Paul, MN; Minnesota Department of Health, St. Paul, MN; New York Emerging Infections Program and University of Rochester Medical Center, Rochester, New York; New York Emerging Infections Program, Rochester, New York; Vanderbilt University Medical Center, Nashville, Tennessee; Vanderbilt University Medical Center, Nashville, Tennessee; Vanderbilt University Medical Center, Nashville, Tennessee; Division of Healthcare Quality Promotion, Centers for Disease Control and Prevention, Atlanta, GA; Centers for Disease Control & Prevnetion, Atlanta, GA; U.S. Centers for Disease Control and Prevention, Atlanta, Georgia

## Abstract

**Background:**

Nasal decolonization with mupirocin has been noted in professional society guidance since 2014 as an optional measure to prevent methicillin-resistant *Staphylococcus aureus* (MRSA) infection in certain high-risk settings or patients (i.e., intensive care unit [ICU] or undergoing high-risk surgery). Because of concerns about emergence of resistance, we evaluated trends in mupirocin resistance among MRSA isolates from invasive infections and associations between healthcare exposures and resistance.

**Figure 1.**
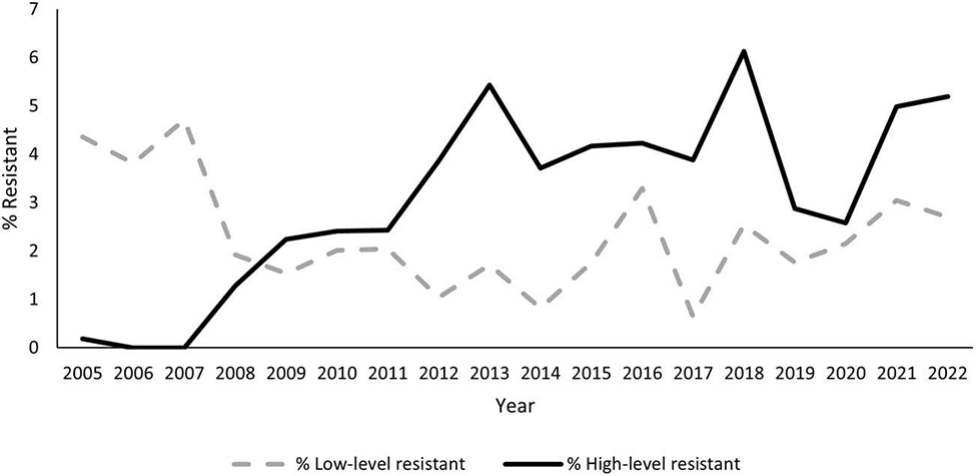
Percentage of methicillin-resistant Staphylococcus aureus isolates from invasive infections with high-level and low-level mupirocin resistance by year, CDC Emerging Infections Program surveillance, 2005-2022

**Methods:**

Isolates from the Emerging Infections Program active, population-based surveillance for invasive MRSA were submitted to CDC from a convenience sample of cases during 2005-2022. A case was MRSA isolated from a normally sterile body site in a surveillance area resident. Isolates were tested using reference broth microdilution and were considered mupirocin susceptible if the minimum inhibitory concentration (MIC) was ≤ 4µg/mL, low-level resistant (LLR) if MIC ≥ 8µg/mL and ≤ 256µg/mL, or high-level resistant (HLR) if MIC > 256µg/mL. Annual proportions of LLR and HLR isolates were calculated. Trends were assessed using the Cochran-Armitage test. For 2019-2022 isolates, healthcare exposures of cases with HLR vs. susceptible isolates were compared.

**Results:**

During 2005-2022, CDC tested 11,451 MRSA isolates for mupirocin resistance, representing 28.5% of total MRSA cases. Annual HLR isolates ranged from 0 – 6.1% (Figure). During 2005-2013, HLR isolates increased from < 1% to 5.4% (*P*< .001); during 2014-2022, HLR isolates ranged between 2.6%-6.1% with no significant trend (*P*=0.55). Cases with HLR (n=68) vs. susceptible (n=1,648) isolates during 2019-2022 did not have significantly different healthcare exposures, including: surgery ≤ 90 days (*P*=0.55) or ≤ 1 year prior (*P*=0.15), hospitalization ≤ 1 year prior (*P*=0.91), ICU care or presence of a central line ≥ 2 days prior (*P*=0.43 and *P*=0.53, respectively), or chronic hemodialysis (*P*=0.34).

**Conclusion:**

Mupirocin resistance among invasive MRSA isolates did not increase significantly after the 2014 guidance and was not associated with selected healthcare exposures. Continued monitoring is needed to assess possible changes in mupirocin resistance as practices and additional strategies for nasal decolonization are evaluated.

**Disclosures:**

William Schaffner, MD, Abbott Dignostics: Honoraria

